# S75. MALADAPTIVE SOCIAL-EVALUATIVE AND SELF-BELIEFS INFORMING THE RELATION BETWEEN PARANOIA AND SOCIAL ANXIETY

**DOI:** 10.1093/schbul/sby018.862

**Published:** 2018-04-01

**Authors:** Alexandra Andrea, LeeAnn Shan, Cristina Garcia, Christina Savage, Melanie Bennett, Jack Blanchard

**Affiliations:** 1 University of Maryland; 2 University of Maryland School of Medicine

## Abstract

**Background:**

Paranoid delusions are reported among those with positive symptoms of schizophrenia (Bentall et al., 2009), those at high-risk for the development of psychosis (Addington et al., 2015; Salokangas et al., 2016), and in the general population (i.e., Freeman et al., 2005). Paranoia is related to functional impairment in multiple domains (i.e. Pinkham et al., 2016; McGurk et al., 2013). Given the presence of paranoia at both clinical and non-clinical levels, paranoia may be best conceptualized dimensionally. Further, robust relations between paranoia and social anxiety suggest that the two may exist together on one spectrum, with paranoia reflecting the most extreme end of this continuum (i.e. Gilbert, Boxall, Cheung, & Irons, 2005; Lim, Rodebaugh, Zyphur & Gleeson, 2016).

Evidence suggests that several factors may influence the development of paranoia, including social cognitive biases such as diminished trust, increased hostility, and increased tendency to blame others (Pinkham et al., 2014). These types of social cognitive biases do not seem to be unique to paranoia and are also observed among those with social anxiety (i.e., Green & Phillips, 2004). Additional research suggests that paranoia and social anxiety may share other relevant attributes, such as need for approval from others (Rector, 2004), desire for closeness (Lim, Rodebaugh, Zyphur & Gleeson, 2016), and worry about and expectation of social rejection (Freeman, 2014). Despite literature to support overlap between paranoia and social anxiety, there is less research examining whether core beliefs of social anxiety contribute to the development and maintenance of paranoia. Specifically, core beliefs of social anxiety include (1) conditional beliefs: negative self-appraisals dependent on performance (2) unconditional beliefs: negative self-appraisals independent of behavior, and (3) high-standard beliefs: the attribution of self-worth based on performance (Wong, Moulds & Rapee, 2014). There is a need for research to determine the association between paranoia and beliefs related to social anxiety.

The aim of the current study is to identify whether cognitions thought to be central to social anxiety are also related to paranoia in a sample of those with psychosis. We hypothesize that (1) social anxiety and paranoia will be related, and (2) heightened maladaptive self-beliefs of social anxiety will be associated with increased paranoia.

**Methods:**

We will use the Social Interaction Anxiety Scale (Heimberg, Mueller, Holt, Hope & Liebowitz, 1992) to measure social anxiety symptomatology. We will use the Self-Beliefs Related to Social Anxiety Scale (Wong, Moulds & Rapee, 2014) including three subscales: conditional beliefs, unconditional beliefs, and high standard beliefs, to quantify cognitions of social anxiety. To measure paranoia, we will use the Green Paranoid Thoughts Scale (Green et al., 2008).

**Results:**

Preliminary analyses (N = 14) indicate that the social anxiety is robustly related to paranoia (r = 0.59, p < 0.05). Unconditional beliefs were related to paranoid thoughts (r = 0.62, p < 0.05) and paranoia was moderately correlated to conditional beliefs (r = 0.35, p = 0.24) and total self-beliefs related to social anxiety scores (r = 0.39, p = 0.19), but these relations were not statistically significant. Additional data will be available at the time of presentation.

**Discussion:**

Results of this research will both inform the development of paranoia and allow for a better understanding of the relation between paranoia and social anxiety. Specifically, cognitive mechanisms underlying their relation will be illuminated.

